# Area deprivation and the prevalence of type 2 diabetes and obesity: analysis at the municipality level in Germany

**DOI:** 10.1186/1471-2458-14-1264

**Published:** 2014-12-13

**Authors:** Nina Grundmann, Andreas Mielck, Martin Siegel, Werner Maier

**Affiliations:** Helmholtz Zentrum München – German Research Center for Environmental Health (GmbH), Institute of Health Economics and Health Care Management, Neuherberg, Germany; Bavarian Health and Food Safety Authority, Erlangen, Germany; Berlin Centre of Health Economics Research (BerlinHECOR), Department of Health Care Management, Technische Universität Berlin, Berlin, Germany

**Keywords:** Area deprivation, Type 2 diabetes, Obesity, Social inequalities, Municipalities, Germany

## Abstract

**Background:**

The objective of this study was to analyse the association between area deprivation at municipality level and the prevalence of type 2 diabetes (T2D) and obesity across Germany, controlling for individual socioeconomic status (SES).

**Methods:**

The analyses are based on a large survey conducted in 2006. Information was included from 39,908 adults aged 20 years or above. Area deprivation was assessed using the German Index of Multiple Deprivation (GIMD) at municipality level. About 4,700 municipalities could be included and assigned to a deprivation quintile. Individual SES was assessed by income and educational level. Multilevel logistic models were used to control for individual SES and other potential confounders such as age, sex and physical activity.

**Results:**

We found a positive association of area deprivation with T2D and obesity. Controlling for all individual-level variables, the odds ratios for municipalities in the most deprived quintile were significantly increased for T2D (OR 1.35; 95% CI 1.12–1.64) as well as for obesity (OR 1.14; 95% CI 1.02–1.26). Further analyses showed that these associations were relatively similar for both men and women.

**Conclusions:**

Based on a nationwide dataset, we were able to show that area deprivation at municipality level is significantly associated with the prevalence of T2D and obesity. It will be important to focus preventive efforts on very deprived municipalities.

## Background

It is well known that health is associated with individual socioeconomic status (SES) and also with area deprivation, and the same seems to be true for type 2 diabetes (T2D) and obesity [1–4]. Concerning the association with area deprivation, a number of studies have been published from the UK [1, 2, 5–7], but there are also studies from France [8], Spain [9], Italy [10], Australia [11] and the US [4]. Most of them show that living in deprived regions is associated with an increased risk of diabetes and obesity. However, only a few investigate whether this association persists after controlling for individual-level SES [4, 5, 8, 11], and all of these studies were confined to specific regions of their countries.

Encouraged by increasing international discussion about area deprivation and health, this topic is steadily gaining more attention in Germany as well. There are already a number of studies on differences in diabetes mellitus or obesity prevalence according to individual SES [12, 13]. The discussion on potential associations with area deprivation has just started [14], however, and the results indicate that area deprivation is important for the prevalence of T2D and obesity too [15, 16]. These studies have some important drawbacks: either they are confined to just a few study regions [15] or the regions compared are relatively large (i.e. the analysis has been conducted at the district level) [16]. It has been discussed repeatedly that the modifiable areal unit problem (MAUP) may play an important role, i.e. that smaller areas may give more significant results than larger ones [17]. This is why we conducted a study that could add a new step, using data from a large nationwide survey that are available on a relatively small regional scale (i.e. municipalities) and controlling for individual SES in multilevel analyses.

There is wide agreement that the increasing prevalences of T2D and obesity are major public health problems. In Germany, the number of adult patients with T2D is currently estimated to be around 4.5 million, resulting in a prevalence of about 7% [18]; these figures include the diagnosed cases only (i.e. undiagnosed cases would have to be added). Also, T2D is associated with severe health consequences. People suffering from T2D have a two- to four-fold higher risk of developing coronary heart disease [19]. Their life expectancy is substantially reduced compared with other people in the same age group. Just looking at men from the lower income group, for example, a recent study from Germany showed that life expectancy is reduced by about 8 years [20].

Thus, this study focuses on the relatively small-scale municipality level and the hypothesis that the prevalences of T2D and obesity both increase with increasing area deprivation, even after controlling for individual-level SES using multilevel modelling. Obesity is included in our analyses for two reasons. First, obesity is a well-established risk factor for T2D and should be included in multivariate analyses where T2D is the dependent variable. Second, multivariate analyses will be added with obesity as the dependent variable.

## Methods

### Study population

Individual-level data were drawn from the TNS Health Care Access Panel (HCAP) which consists of a number of cross-sectional surveys conducted across Germany. The HCAP was based on a large German household sample and was developed as an alternative to face-to-face or telephone health survey interviews in order to estimate the prevalence or incidence rates of health-relevant variables. The households were not randomly recruited; participation was voluntary. Underrepresented cells, e.g. age groups or regions, were complemented. More detailed information on the HCAP data has been described previously [21].

HCAP data have already been used in other scientific publications [22–24]. They are well suited to small-scale regional comparisons, as they include the official municipality keys which allow unambiguous identification of the respective municipality. We used the survey conducted in 2006, comprising n = 60,555 participants, as it included an explicit question on T2D. All data were collected by mailed questionnaire and are thus based on self-report. The response rate was 60% [23]. A number of participants had to be excluded. First, the analyses were restricted to participants aged 20 years or above, as information on educational level and income is more meaningful for adults, and as the risk of T2D is very low in younger age groups. This reduced the number of participants from n = 60,555 to n = 49,004. Second, municipality keys could not be assigned to n = 4,954 participants. This was mainly the case for Rhineland-Palatinate, i.e. a relatively small federal state in the south-west of Germany. Third, we removed all cases with other or unknown types of diabetes (i.e. type 1, unknown type, missing or no specification of type; n = 426), as well as all cases with missing information on body mass index (BMI) (n = 2,769). Fourth, we also removed those with missing information concerning equivalent income (n = 576) and with missing information or no specification concerning physical activity (n = 1,097), thus performing a complete case analysis. The final dataset includes information from n = 39,908 participants.

### Ethics and consent statement

Ethics approval for this retrospective study was not mandatory as no medical research involving human subjects was conducted. Participation in the TNS HCAP was entirely voluntary and no costs arose for the participants. Data were obtained through a self-administered survey and the participants received written information on the subsequent use of the data for research purposes (commercial and non-commercial) together with the mailed questionnaires.

### Individual-level variables

The question on diabetes in the 2006 survey reads ‘Are you suffering from diabetes?’ and gives four categories: ‘Yes, from type 1 diabetes (juvenile diabetes); Yes, from type 2 diabetes (adult-onset diabetes); Yes, but type of diabetes unknown; No’. Excluding type 1 diabetes and ‘diabetes type unknown’, the resulting dichotomous variable was ‘T2D vs. no T2D’.

Obesity was assessed by BMI (kg/m^2^). Five groups of BMI were differentiated [25]: underweight (BMI < 18.5); normal weight (18.5 ≤ BMI < 25); pre-obesity (25 ≤ BMI < 30); moderate obesity (30 ≤ BMI < 35); severe obesity (BMI ≥ 35). The groups ‘underweight’ (less than 2%) and ‘normal weight’ were combined to serve as a reference category. In the analyses with obesity as the dependent variable, obesity was defined as a dichotomous variable (obese vs. not obese) according to the WHO guidelines for obesity (BMI ≥ 30 kg/m^2^).

Individual SES was assessed by equivalent income, using the definition proposed in the Luxembourg Income Study [26] (net household income/household size^0.36^). Four income groups were differentiated: below 60% of the median income (i.e. ‘poor’); ≥ 60% and < 100%; ≥ 100% and < 150%; ≥ 150% of the median. Additional analyses (not reported below) have been conducted with educational level instead of equivalent income. In our analyses, the associations with T2D and obesity were stronger for equivalent income than for educational level. In order to provide more conservative estimates for the independent variable ‘area deprivation’, we finally opted to include equivalent income in the multilevel analyses presented below.

Three other individual-level variables were included as well, as they could have a strong influence on the risk of diabetes and obesity [12, 27, 28]: sex, age (five categories: 20–39, 40–49, 50–59, 60–69 and 70 years and over) and physical activity, as assessed by the question: ‘Are you taking exercise regularly – at least once a week?’, with the categories ‘Yes’ or ‘No’.

### Area deprivation

Area deprivation of the municipalities where the participants lived was assessed by the German Index of Multiple Deprivation (GIMD). This index has been developed recently, based on the Indices of Multiple Deprivation (IMD) generated by Noble and colleagues [29]. The GIMD includes demographic, socioeconomic and environmental characteristics related to seven different domains of deprivation (i.e. income, employment, education, municipal revenue, social capital, environment, security) [14, 15]. The index already showed significant associations with T2D, reported first in a previous study confined to five regional surveys [15], and then in a study with health survey data from the Robert Koch Institute (i.e. the federal institution for disease control and prevention in Germany) [16]. The second analysis was restricted to the regional scale of districts, which are mostly much larger than municipalities. Municipalities are the lowest level of administrative division in Germany and cover a wide range of population size, including small rural municipalities with less than 100 inhabitants up to cities with more than one million inhabitants such as Munich or Berlin [15]. In Germany in December 2006, there were n = 439 districts, but n = 9,620 municipalities (comprising single municipalities and associations of municipalities). At that time, the total population of Germany was 82.3 million living in 16 federal states of varying population size (ranging from almost 0.7 to 18 million) and also differing in land area and economic power [30].

The GIMD has also been used in analyses with other health-related outcomes, such as antibiotic prescriptions, hip and knee joint replacements and cancer survival [31–33]. To date, it is the only available area deprivation index covering the whole of Germany. GIMD scores were calculated for all 9,620 municipalities in Germany [15] and divided into quintiles, with the first quintile (Q1) including the least deprived and the fifth quintile (Q5) including the most deprived municipalities. Our dataset included 4,643 municipalities, well spread across the whole of Germany.

### Statistical analyses

In the bivariate analyses, the Cochran–Armitage test for trend was used to test whether the prevalence of T2D and obesity increases significantly with increasing deprivation. Logistic multilevel analyses were conducted for T2D and obesity separately (level 1: individuals; level 2: municipalities). Different models are shown in order to demonstrate how the odds ratios (OR) and 95% confidence intervals (95% CI) for deprivation at municipality level change when different sets of variables are included. The analyses were conducted with the statistical software SAS 9.2 (SAS Institute Inc., Cary, NC, USA), using the GLIMMIX procedure for the multilevel analyses with a maximum likelihood estimation based on Laplace approximation.

## Results

Table 1 shows the distribution of the variables. The dataset comprised 39,908 people aged 20 years or above; about 18% of them lived in municipalities with the highest area deprivation (quintile 5).

**Table 1:** **Univariate distribution of the variables**

	n	(%)
Total	39908	(100)
**Dependent variables**		
Type 2 diabetes		
Yes	2269	(5.7)
No	37639	(94.3)
Obesity		
Yes	7447	(18.7)
No	32461	(81.3)
**Independent variables**		
Sex		
Men	18263	(45.8)
Women	21645	(54.2)
Age (years)		
20–39	15211	(38.1)
40–49	8438	(21.1)
50–59	8159	(20.4)
60–69	7044	(17.7)
> 70	1056	(2.7)
BMI (kg/m^2^)		
< 25	18220	(45.7)
25 to < 30	14241	(35.7)
30 to < 35	5295	(13.3)
≥ 35	2152	(5.4)
Equivalent income (% of median income)		
< 60	6687	(16.8)
≥ 60 to ≤ 100	13236	(33.2)
> 100 to ≤ 150	13238	(33.2)
> 150	6747	(16.9)
Physical activity		
Yes	20099	(50.4)
No	19809	(49.6)
Area deprivation (GIMD)^a^		
Quintile 1 (least deprived)	5450	(13.7)
Quintile 2	8144	(20.4)
Quintile 3	9216	(23.1)
Quintile 4	9761	(24.5)
Quintile 5 (most deprived)	7337	(18.4)

^a)^GIMD, German Index of Multiple Deprivation.

The bivariate associations of municipal deprivation with T2D and obesity are shown in Table 2. The prevalence of T2D clearly increased with increasing deprivation for both men and women, and the Cochran–Armitage test for trend indicated that this increase was highly significant. The same picture could be seen for obesity. The differences between Q1 and Q5 were substantial: the risk of T2D among men was about 1.7-fold (8.9% vs. 5.3%) higher in the most deprived quintile, and the risk of obesity among women was 1.3-fold (21.8% vs. 16.9%) higher.

**Table 2:** **Bivariate association of type 2 diabetes or obesity and area deprivation**

	Area deprivation						
	Quintile 1^a^ % (n)	Quintile 2 % (n)	Quintile 3 % (n)	Quintile 4 % (n)	Quintile 5^b^ % (n)	p-values [p for trend]^c^	Total % (n)
Type 2 diabetes							
Men	5.3 (133)	6.6 (248)	6.2 (262)	7.2 (321)	8.9 (295)	p < 0.0001	6.9 (1259)
Women	3.3 (98)	3.9 (169)	4.8 (238)	5.1 (273)	5.8 (232)	p < 0.0001	4.7 (1010)
Total	4.2 (231)	5.1 (417)	5.4 (500)	6.1 (594)	7.2 (527)	p < 0.0001	5.7 (2269)
Obesity							
Men	16.4 (412)	16.4 (621)	18.0 (759)	18.7 (830)	19.9 (663)	p < 0.0001	18.0 (3285)
Women	16.9 (496)	17.7 (771)	18.9 (944)	20.3 (1078)	21.8 (873)	p < 0.0001	19.2 (4162)
Total	16.7 (908)	17.1 (1392)	18.5 (1703)	19.6 (1908)	20.9 (1536)	p < 0.0001	18.7 (7447)

^a)^Quintile 1 ‘lowest deprivation’.

^b)^Quintile 5 ‘highest deprivation’.

^c)^Cochran–Armitage test for trend.

The multilevel analyses for T2D as the dependent variable can be summarised in the following way (Table 3). After controlling for age and sex alone, the diabetes risk in municipalities with the highest deprivation (Q5) was about 1.7 times higher than in municipalities with the lowest deprivation (Q1). This association decreased after controlling for the other individual-level variables (i.e. sex, age, BMI, equivalent income, physical activity), but it remained statistically significant for the most deprived quintile Q5 over all models. As expected, the other individual-level variables (e.g. age or physical activity) were also significantly associated with the prevalence of T2D. Separate analyses for men and women (not presented in the table) indicated that the risk of having T2D was slightly stronger among men in the most deprived quintile Q5.

**Table 3:** **Logistic multilevel analysis of the dependent variable ‘type 2 diabetes’ (n = 39,908)**

	Model 1 OR (95% CI)	Model 2 OR (95% CI)	Model 3 OR (95% CI)	Model 4 OR (95% CI)
GIMD^a^				
Q1	1.00	1.00	1.00	1.00
Q2	**1.24** (1.02–1.50)	**1.22** (1.01–1.47)	1.19 (0.98–1.44)	1.18 (0.97–1.42)
Q3	**1.33** (1.10–1.61)	**1.28** (1.06–1.55)	**1.22** (1.01–1.48)	**1.21** (1.00–1.47)
Q4	**1.36** (1.13–1.63)	**1.27** (1.06–1.53)	1.18 (0.98–1.42)	1.16 (0.97–1.40)
Q5	**1.66** (1.37–2.00)	**1.52** (1.25–1.83)	**1.37** (1.13–1.65)	**1.35** (1.12–1.64)
Sex				
Women	1.00	1.00	1.00	1.00
Men	**1.42** (1.30–1.55)	**1.44** (1.31–1.59)	**1.51** (1.38–1.66)	**1.48** (1.35–1.63)
Age (years)				
20–39	1.00	1.00	1.00	1.00
40–49	**2.75** (2.24–3.38)	**2.35** (1.91–2.89)	**2.39** (1.94–2.95)	**2.39** (1.94–2.94)
50–59	**9.11** (7.62–10.88)	**7.15** (5.97–8.56)	**7.38** (6.16–8.85)	**7.36** (6.14–8.82)
60–69	**15.91** (13.37–18.93)	**13.32** (11.16–15.89)	**13.54** (11.34–16.16)	**13.67** (11.45–16.32)
> 70	**21.00** (16.73–26.37)	**18.67** (14.80–23.55)	**18.86** (14.94–23.80)	**18.71** (14.82–23.61)
BMI^b^				
< 25		1.00	1.00	1.00
< 30		**1.93** (1.69–2.19)	**1.90** (1.67–2.17)	**1.88** (1.65–2.14)
< 35		**4.50** (3.92–5.16)	**4.33** (3.77–4.97)	**4.17** (3.63–4.79)
≥ 35		**10.15** (8.66–11.90)	**9.49** (8.09–11.14)	**9.00** (7.66–10.55)
Income^c^				
1 (> 150)			1.00	1.00
2			**1.36** (1.16–1.59)	**1.34** (1.14–1.57)
3			**1.51** (1.29–1.77)	**1.47** (1.25–1.72)
4 (< 60)			**2.07** (1.75–2.46)	**2.00** (1.68–2.37)
PA^d^				
Yes				1.00
No				**1.27** (1.15–1.40)
V_A_^e^	0.314	0.249	0.253	0.245
SE^f^	0.069	0.063	0.064	0.064

Bold type = significant.

^a)^GIMD, German Index of Multiple Deprivation (Quintile 5: ‘most deprived’).

^b)^BMI, body mass index (kg/m^2^).

^c)^Equivalent income (% of median income).

^d)^PA, physical activity.

^e)^V_A_, area-level variance (municipalities).

^f)^SE, standard error.

Turning to the dependent variable ‘obesity’, very similar associations with area deprivation at the municipality level could be seen here as well (Table 4). The risk of being obese increased with increasing area deprivation, and it remained statistically significant for the most deprived municipalities (see quintiles 4 and 5) even after controlling for all individual-level variables (i.e. sex, age, equivalent income, physical activity). Also, the other individual-level variables were significantly associated with the prevalence of obesity. In separate analyses for men and women (not presented in the table), the associations were very similar in both groups, but slightly stronger among women.

**Table 4:** **Logistic multilevel analysis of the dependent variable ‘obesity’ (n = 39,908)**

	Model 1 OR (95% CI)	Model 2 OR (95% CI)	Model 3 OR (95% CI)
GIMD^a^			
Q1	1.00	1.00	1.00
Q2	1.07 (0.97–1.19)	1.05 (0.95–1.16)	1.03 (0.93–1.14)
Q3	**1.14** (1.03–1.26)	1.09 (0.98–1.20)	1.07 (0.96–1.18)
Q4	**1.24** (1.12–1.37)	**1.14** (1.03–1.26)	**1.10** (1.00–1.22)
Q5	**1.32** (1.19–1.47)	**1.18** (1.06–1.31)	**1.14** (1.02–1.26)
Sex			
Women	1.00	1.00	1.00
Men	**0.91** (0.86–0.96)	**0.94** (0.90–0.99)	**0.89** (0.85–0.94)
Age (years)			
20–39	1.00	1.00	1.00
40–49	**1.54** (1.43–1.66)	**1.57** (1.46–1.69)	**1.58** (1.46–1.70)
50–59	**2.15** (2.00–2.30)	**2.24** (2.09–2.40)	**2.20** (2.05–2.36)
60–69	**2.00** (1.85–2.15)	**2.01** (1.87–2.17)	**2.06** (1.91–2.22)
> 70	**1.77** (1.51–2.08)	**1.77** (1.51–2.07)	**1.71** (1.45–2.00)
Income^b^			
> 150		1.00	1.00
≤ 150 to > 100		**1.43** (1.32–1.56)	**1.37** (1.25–1.49)
≤ 100 to ≥ 60		**1.76** (1.62–1.92)	**1.61** (1.47–1.75)
< 60		**2.10** (1.91–2.31)	**1.87** (1.70–2.06)
Physical activity			
Yes			1.00
No			**2.00** (1.90–2.11)
V_A_^c^	0.104	0.085	0.076
SE^d^	0.021	0.020	0.019

Bold type = significant.

^a)^GIMD, German Index of Multiple Deprivation (Quintile 5: ‘most deprived’).

^b)^Equivalent income (% of median income).

^c)^V_A_, area-level variance (municipalities).

^d)^SE, standard error.

## Discussion

Our main results were that the prevalences of T2D and obesity both increase with increasing area deprivation at the municipality level, and that the prevalence in the most deprived municipalities is clearly higher compared with the least deprived municipalities, even after controlling for individual SES in multilevel analyses.

Some limitations have to be taken into account. First, the response rate was 60%. This is well within the range of other health surveys, but there are still many non-responders, and a non-response analysis has not been conducted. We assume that non-responders mostly come from low SES groups [34] and from highly deprived municipalities. This might underestimate the reported associations. Second, all individual-level variables are based on self-report. It is difficult to assess the potential bias introduced by self-reporting. It is well known, for example, that many people with T2D are not aware of their condition [35]. In Germany, all population groups have good access to physicians. Communication with the physician could differ by SES, of course, but apparently this hardly affects the diagnosis of T2D [12]. Third, physical activity could just be assessed in a rather crude way. Fourth, municipalities are the smallest administrative division in Germany for which official data are available. However, the size of municipalities is quite heterogeneous, ranging from less than 100 inhabitants up to cities with more than one million. This large variation could be a problem, as the accuracy of classifying individuals by area deprivation could depend on the size of the regions compared. Fifth, we assume that the association between area deprivation and diabetes and obesity could be even stronger if smaller areas such as neighbourhoods were compared, as misclassification might be reduced, but this was not possible in the present analysis. Lastly, the results cannot be interpreted in a causal way, as they are based on cross-sectional data.

We believe that our study could be a valuable contribution to the discussion on area deprivation and health. It is based on a large nationwide dataset and uses an established area deprivation measure for Germany. Also, the results are quite stable. To date, there have been only a few comparable studies. Looking at the dependent variable ‘type 2 diabetes’, two of these studies come from Germany, but they are restricted to five German study regions [15] or they are looking at districts [16]. As mentioned above, districts are mostly much larger than municipalities. On 31 December 2011, the mean population size of the municipalities was 7,201 inhabitants (median: 1,663) [36], whereas the mean population size of the districts was 203,591 inhabitants (median: 149,032) [37]. It is important to point out that, in the case of T2D, our model 1 (i.e. adjusted for sex and age) provided somewhat stronger odds ratios (OR) at the municipality level than the corresponding model at the district level reported in a previous study [16]. This may be related to the modifiable areal unit problem (MAUP), as already mentioned in the introduction [17], and underlines the importance of including small area units in these analyses. However, a direct comparison between these two studies was possible only for these two basic models.

Turning to studies from other countries, an analysis from the British Women’s Heart and Health Study shows a positive association between area deprivation and the prevalence of diagnosed T2D that persisted after adjusting for individual life-course SES [5]. A study from Scotland shows that T2D is becoming more and more concentrated in deprived regions [6]. Another paper from Scotland adds the information that the incidence of T2D in deprived regions is reduced if these areas are surrounded by less deprived regions [1]. Similar results have also been reported for the dependent variable ‘obesity’. One study comprises data from nine towns in the Czech Republic and Germany, focusing on the neighbourhood level [38]. Another study focuses on different neighbourhoods within a city in the Netherlands [39]. Other studies come from Sweden [40], France [41], Australia [11] and the US [4]. They all show that neighbourhood or municipality deprivation contributes to obesity, even after controlling for individual-level variables.

Our results fit well into this picture, adding further evidence from a large dataset covering the whole of Germany. Also, we were able to control for individual-level SES in multilevel analyses, adding further support to the hypothesis that there is a regional-level effect over and above the individual-level effect. Most of the studies mentioned above are also based on multilevel analyses [4, 8, 11, 15, 42], but they are all restricted to limited study areas within their countries and do not have a nationwide approach. Compared with other studies [4, 11, 15, 42], we were able to conduct our multilevel analyses based on a large number of participants (n = 39,908) and a large number of areas (n = 4,643), looking at both T2D and obesity.

To date, the potential causal pathways behind these regional-level associations have not been understood in any detail. Whereas individual SES may have a more direct influence on health (e.g. by providing individual financial or educational resources for a healthier lifestyle), area-level deprivation may act through a network of collective infrastructural resources such as resources for recreational activities, the availability of healthy food and medical care [15, 43]. Regional traditions can influence individual behavioural norms and attitudes and thus affect health behaviour [15, 44]. People living in more deprived areas could be exposed to more adverse environmental conditions, such as traffic noise and air pollution, and higher crime rates could result in higher levels of chronic stress [8, 45]. Also, higher levels of chronic stress are associated with abdominal obesity and with the development of insulin resistance [46]. Psychological factors could well contribute to the association between area deprivation and health [44]. Prevention strategies should not focus only on the behaviour of people but also on the conditions in which they live. Considering area deprivation indicators when implementing prevention measures is essential in order to make them more effective.

## Conclusions

The empirical finding that municipal deprivation is associated with T2D and obesity could be of great public health importance. It could be the basis for directing more public health resources aimed at reducing the risk of diabetes and obesity towards deprived municipalities. Much is known about potential ways of reducing the risk of diabetes and obesity. It would be important to focus these efforts more on those municipalities most in need.
